# Supplementary material for: Unlocking ultrafast hot hole transport in transition metal oxides governed by the nature of optical transitions
Source: Nat Commun. 2025 Nov 14;16:10024. doi: 10.1038/s41467-025-66193-x (PMC12618571; doi:10.1038/s41467-025-66193-x)
Supplement: Supplementary file 1 — Supplementary Information [file 41467_2025_66193_MOESM1_ESM.pdf]

Supplementary Information for

Unlocking Ultrafast Hot Hole Transport in Transition Metal Oxides Governed by the Nature of Optical Transitions

Keming Li[1, †], Yingjie Wang[1, †], Lan Jiang[1,2,3], Guoquan Gao[1], Guanzhao Wen[4], Yan Zhang[5], Xianjie Wang[6], Shuaifeng Lou[5], Mischa Bonn[4], Hai I. Wang[4,7]*, Tong Zhu[1]*

[1]Laser Micro/Nano Fabrication Laboratory, School of Mechanical Engineering, Beijing Institute of Technology, Beijing, China

[2]Beijing Institute of Technology Chongqing Innovation Center, Chongqing, China

[3]Yangtze Delta Region Academy, Beijing Institute of Technology, Jiaxing, China

[4]Max Planck Institute for Polymer Research, Mainz, Germany

[5]State Key Laboratory of Space Power-Sources, Harbin Institute of Technology, Harbin, China

[6]School of Physics, Harbin Institute of Technology, Harbin, China

[7]Nanophotonics, Debye Institute for Nanomaterials Science, Utrecht University, Utrecht, the Netherlands

[†]These authors contributed equally: Keming Li, Yingjie Wang

*E-Mail: h.wang5@uu.nl; tongzhubit@bit.edu.cn

Table of Contents

Supplementary Note 1 1
Supplementary Note 2 2
Supplementary Note 3 3
Supplementary Note 4 4
Supplementary Note 5 9
Supplementary Note 6 10
Supplementary Note 7 11
Supplementary Note 8 13
Supplementary Note 9 14
Supplementary Note 10 16
Supplementary Note 11 17
Supplementary Note 12 19
Supplementary Note 13 21
Supplementary Note 14 23
Supplementary Note 15 25
Supplementary Note 16 26
Supplementary Note 17 29
Supplementary Note 18 30
Supplementary Note 19 33
Supplementary references 34

Supplementary Note 1

Film Characterization

The Raman spectroscopy of the Co_3O_4 films (Supplementary Figure 1) reveals distinct Raman peaks at 681 cm^{-1}, 618 cm^{-1}, 521 cm^{-1}, and 482 cm^{-1}, which are consistent with the findings reported in prior literature[1, 2], thereby confirming the high purity of the film samples.

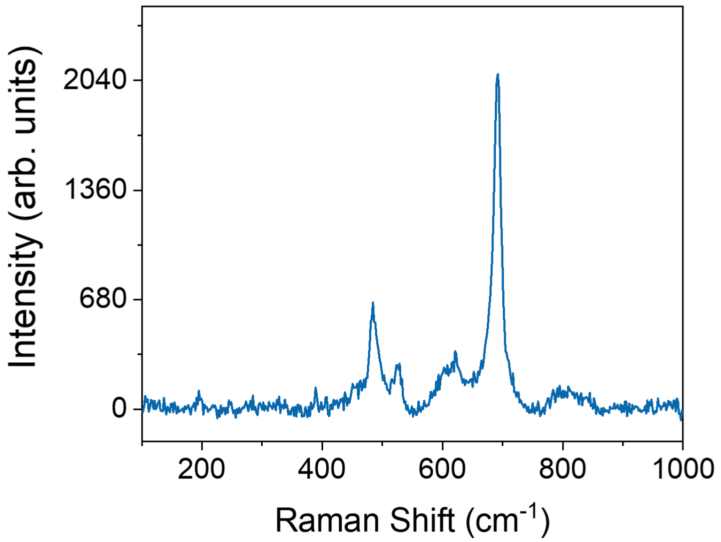

Supplementary Figure 1: Raman spectra of Co_3O_4 thin film.

Cross-sectional SEM images of the Co_3O_4, α-Fe_2O_3, and Cu_2O films used in this study are shown in Supplementary Figure 2. The typical thicknesses are approximately 60 nm, 30 nm, and 220 nm, respectively.

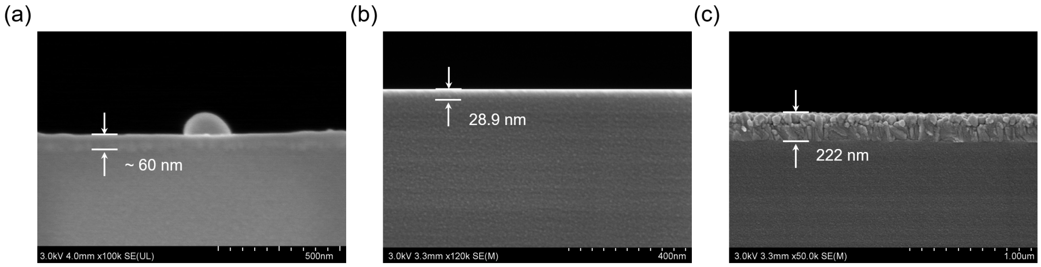

Supplementary Figure 2: SEM cross-sections of the (a) Co_3O_4, (b) α-Fe_2O_3, and (c) Cu_2O thin films used in this study.

Supplementary Note 2

Steady State Absorption of Co_3O_4 Thin Film

The absorption spectra of Co_3O_4 film were measured using Agilent Cary 7000 UV-Visible-infrared spectrophotometer. The steady-state absorption spectra data of the Co_3O_4 film were fitted using a multi-peak Gaussian model and the fitting parameters referring to the assignment by Qiao et al.[3], as shown in Supplementary Table 1.

Supplementary Table 1: Optical transition assignments for Co_3O_4

Peak	Energy (eV)	Intensity (arb. units)	Linewidth (FWHM, eV)
MMT 1	0.87	1.00	0.29
MMT 2	1.04	5.12	0.77
MMT 3	1.64	2.38	0.36
MMT 4	1.84	5.52	0.61
LMCT 1	2.62	25.23	1.05
LMCT 2	3.70	41.56	1.30

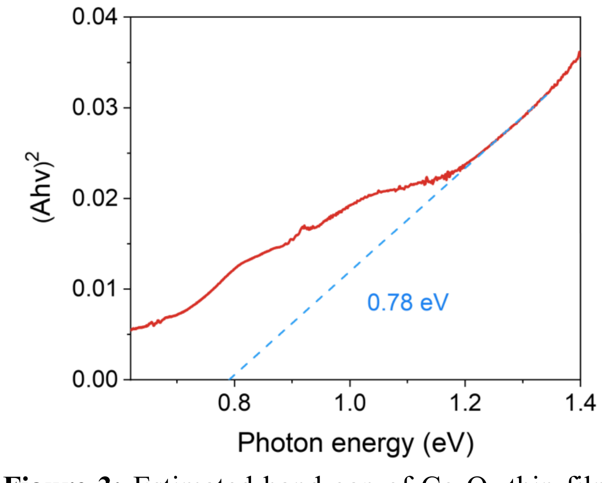

Supplementary Figure 3: Estimated band gap of Co_3O_4 thin film obtained by Tauc method.

Supplementary Note 3

Hole-Origin Assignment of the 600 nm Feature in Co_3O_4 TA

The scavenger experiments provide direct evidence for the hole-related origin of the 600 nm feature. In TMOs, methanol is a widely used hole scavenger that rapidly consumes photogenerated holes, whereas $AgNO_3$ serves as a standard electron scavenger. We measured the TA kinetics of Co_3O_4 at 600 nm in three media—water, 10% v/v MeOH, and 2 mM $AgNO_3$—while keeping the pump fluence constant (Supplementary Figure 4). In the presence of MeOH, the 600 nm signal exhibits a markedly faster decay, consistent with the selective depletion of holes. In contrast, no change in the 600 nm dynamics is observed with $AgNO_3$, further confirming that the signal at this wavelength originates from holes.

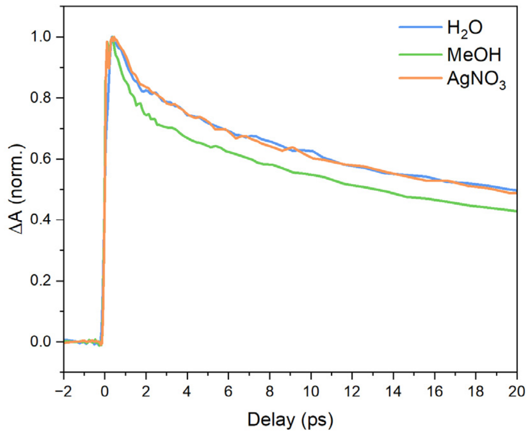

Supplementary Figure 4: TA kinetics of Co_3O_4 at 600 nm in water, with 10 % MeOH (hole scavenger), and with 2 mM $AgNO_3$ (electron scavenger).

In addition, Waegele et al. differentiated the contributions of electrons and holes to the spectral signals through spectroelectrochemical measurements by selectively filling electron states with applied voltage[4]. The PIA near 600 nm is attributed to hole-induced absorption, while the bleach signal at 750 nm is assigned to electron-induced bleaching. Based on these results and considering the band structure of Co_3O_4, we attribute the PIA at 600 nm to electron transitions from the O 2p orbitals to the top of the valence band.

Supplementary Note 4

Blue Shift of Transient Absorption (TA) of Co_3O_4

Notably, this PIA signal exhibits a blue shift within approximately 1 ps. Generally, Coulomb-induced bandgap renormalization, thermal effect, or the formation of localized states (Including defect trapping or self-trap carrier) could account for such spectral features.

Coulomb-induced bandgap renormalization (BGR) could, in principle, lead to such spectral shifts, with the magnitude expected to scale with the density of injected carriers. However, our TA experiments reveal no pump-fluence dependence of the blue shift (Supplementary Figure 5), nor do they show any further spectral shifts within the time window up to 5000 ps, as would be expected for bandgap recovery following Coulomb-induced BGR. Moreover, the TA spectra around 600 nm exhibit neither a noticeable change in the magnitude of the spectral shift nor any additional spectral broadening across different pump fluences[5, 6]. Given the characteristic fluence dependence of BGR, the absence of such trends strongly suggests that the 600 nm feature does not originate from BGR.

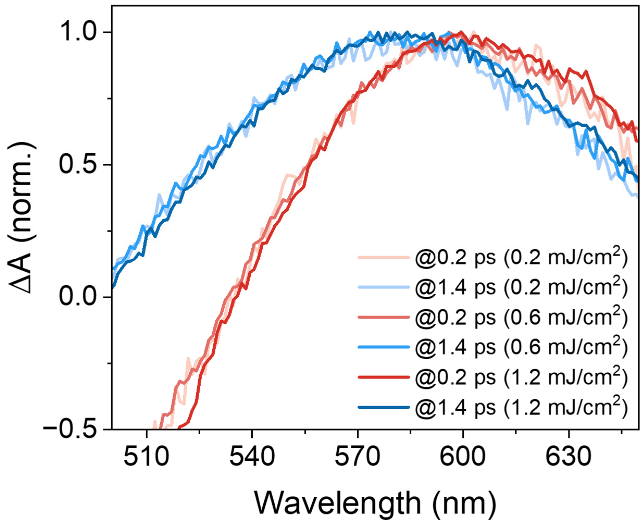

Supplementary Figure 5: In power-dependent TA experiments, the spectra of 600 nm PIA signal before blue shift (0.2 ps) and after blue shift (1.0 ps) were selected to compare the change of blue shift under different pump fluences. Pump wavelength: 700 nm.

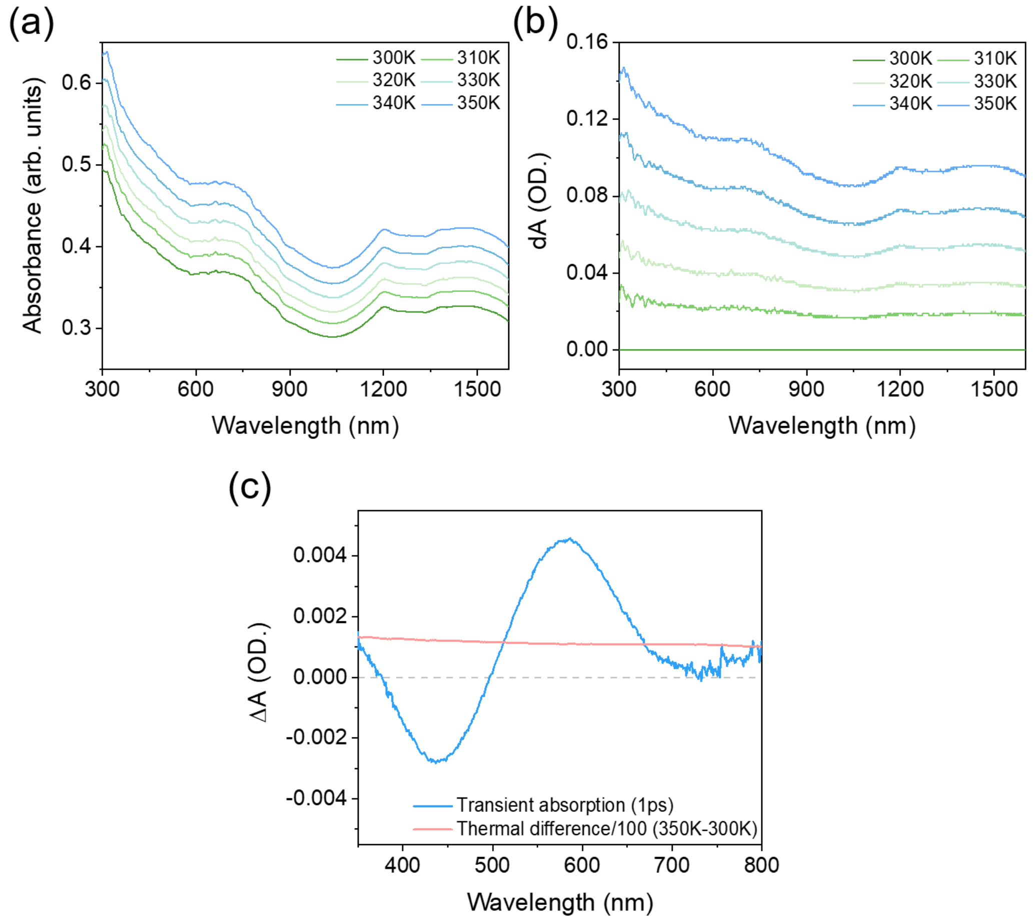

Supplementary Figure 6: (a) Temperature-dependent absorption spectra of Co_3O_4 thin films in the range of 300 K to 350 K. (b) The thermal difference spectrum is obtained by subtracting the absorption spectrum at different temperatures with that at 300 K. (c) Comparison between the dA collected by TA at the delay time of 1 ps and the thermal difference spectrum from the absorption spectra taken at 350 K and 300 K.

Additionally, sample heating induced by illumination can significantly impact the transient spectra of TMOs, introducing noteworthy changes[7, 8]. For instance, as the temperature rises, thermal expansion can alter the semiconductor bandgap and phonon coupling, leading to a broadening of the absorption edge and new spectral features. The blue shift of the PIA signal at 600 nm occurs within 1 ps, which is significantly shorter than the time scale typically associated with thermal effects (greater than 100 ps). However, in order to further rule out the possibility of spectral response caused by thermal effects, the Shimadzu UV-3600IPLUS UV-Visible-infrared spectrophotometer was used to measure the thermal difference spectra in the temperature range of 300 K to 350 K, as shown in Supplementary Figure 6(a). The thermal difference spectrum

indicates that absorption decreases at lower temperatures (Supplementary Figure 6(b)), but the temperature has no significant impact on the line shape of the Co_3O_4 film's TA spectra (Supplementary Figure 6(c)). Therefore, thermal effects are not the primary cause of the blue shift.

Defect trapping could also contribute to spectral shifts, but given the discrete nature of defect energy levels, carrier capture by defects typically does not result in continuous spectral shifts. Moreover, our power-dependent TA measurements showed no variation in decay rates (Supplementary Figure 7), allowing the exclusion of defect trapping.

Different from defect trapping, the formation of polaronic state involves a continuous energy transition. Carriers form small polarons through carrier-phonon interactions, and the accompanying lattice polarization alters their energy. Generally, carrier-phonon coupling in semiconductors manifests within the ultrafast time scale, a phase where electronic and structural states are not in equilibrium.

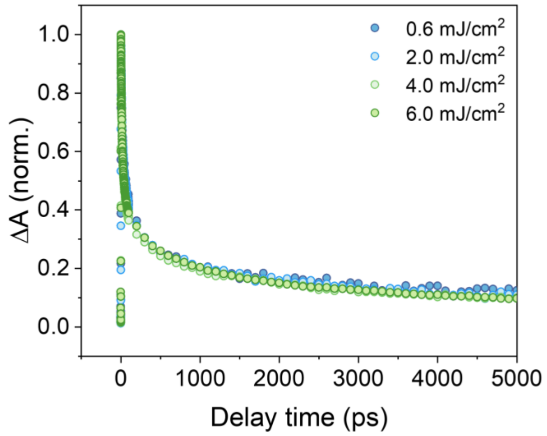

Supplementary Figure 7: TA dynamics corresponding to different excitation fluence at 570 nm.

The apparent spectral shift could also arise from overlap between two positive features near 610 nm and 570 nm, where a faster decay at 600 nm would give the appearance of a blue shift. However, TA kinetics at 570 nm and 615 nm show a concurrent rise and decay, respectively (Supplementary Figure 8), consistent with a state-conversion process rather than fixed-component spectral overlap. Furthermore, the integrated kinetics over 550-650 nm (Figure 3b in the main text) indicate a stable carrier density,

excluding carrier recombination as the cause of signal decay. Considering the energy stabilization associated with polaron formation, we attribute this feature to ultrafast hole self-trapping accompanied by a change in transition energy that shifts the absorption center, an interpretation further supported by our transport measurements.

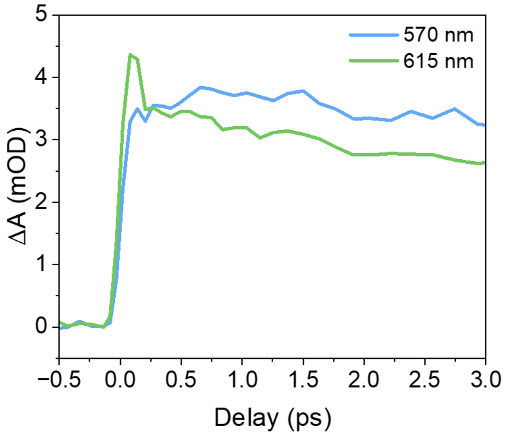

Supplementary Figure 8: TA dynamics of Co_3O_4 at different probe wavelengths.

Based on the above discussion, the dynamics observed in the TA spectra are schematically represented as shown in Supplementary Figure 9.

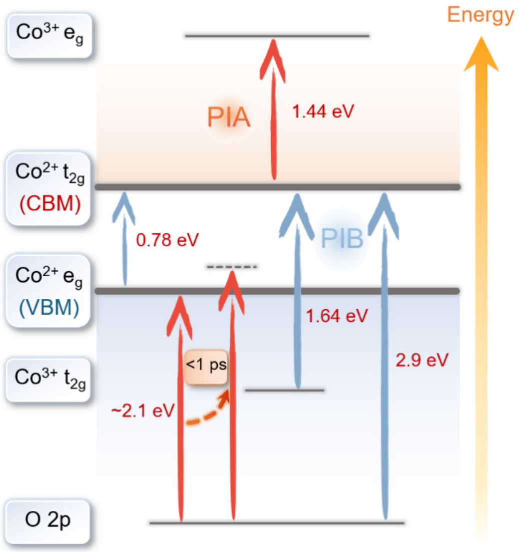

Supplementary Figure 9: Schematic diagram of carrier dynamics in Co_3O_4.

In addition, for all TA measurements, we ensured that the sample response to the

excitation is proportional to the intensity of the pump. This proportionality is crucial for accurately interpreting absorption changes in excited states, as opposed to artifacts caused by nonlinear phenomena such as multi-photon absorption or optical saturation. The relationship between dynamic peak intensity and pump fluence is shown in Supplementary Figure 10.

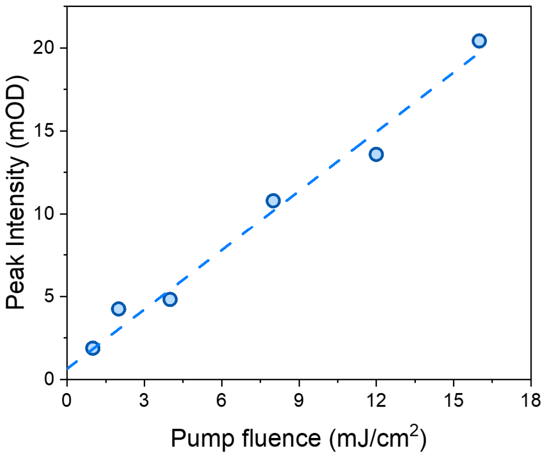

Supplementary Figure 10: Peak signal intensity as a function of the pump fluence. The blue dashed line is a linear fit. The pump wavelength is 700 nm and the probe wavelength is 590 nm.

Supplementary Note 5

Schematic Illustration of the Ultrafast Optical Nanoscopy Setup.

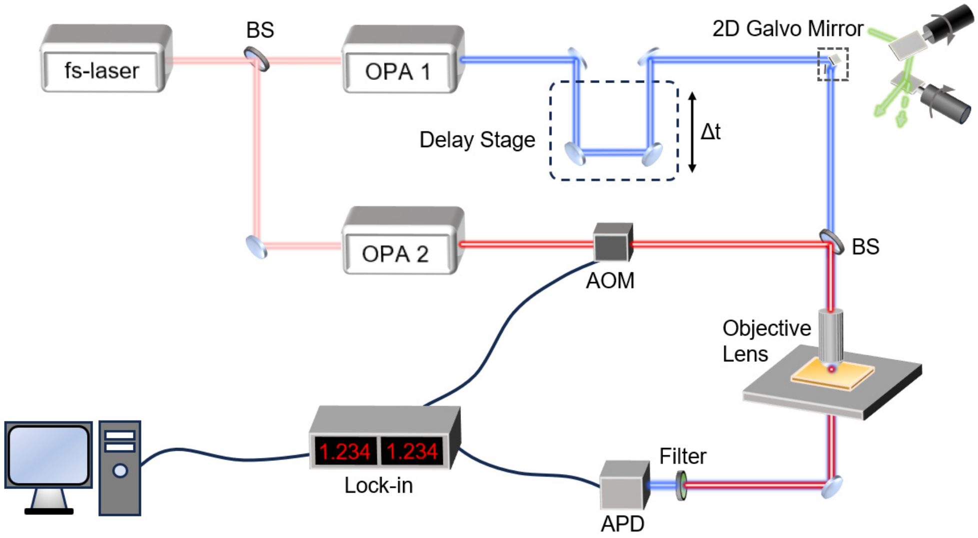

Supplementary Figure 11: Schematic illustration of the ultrafast optical nanoscopy setup[9]. Abbreviations: AOM, acousto-optic modulator; APD, avalanche photodiode; BS, beam splitter; OPA, optical parametric amplifier.

Supplementary Note 6

Effect of Film Thickness on Hot Carrier Diffusion

Our ultrafast optical nanoscopy experiments were conducted in a transmission geometry, where the film thicknesses are considerably smaller than the optical penetration depth under our excitation conditions (typically >300 nm). This ensures that the excitation profile along the film thickness is approximately uniform, and axial field attenuation can be neglected. Consequently, carrier transport can be effectively modeled in the lateral direction, without requiring depth-resolved corrections.

To further confirm that the extracted diffusion behavior is intrinsic and not strongly modulated by thickness-dependent effects (e.g., surface scattering or vertical transport constraints), we repeated the measurements on a much thicker Co_3O_4 film (~50 μm) under identical excitation conditions to those in Figure 2c. As shown in Supplementary Figure 12, the extracted MSD evolution and diffusion constants closely match those of the 60 nm thin film. This consistency across vastly different thickness regimes supports our interpretation that the reported hot carrier diffusion behavior arises from intrinsic material and excitation properties, rather than being an artifact of limited film thickness.

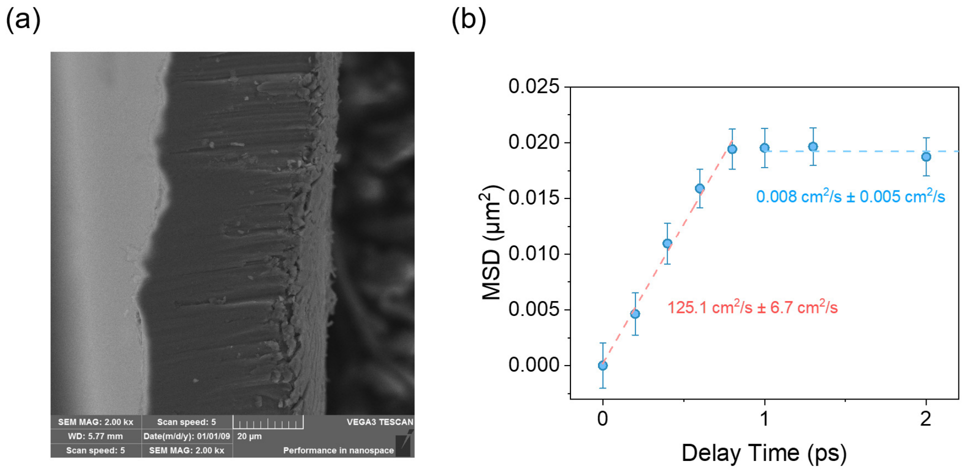

Supplementary Figure 12: (a) SEM cross-sections of thick Co_3O_4 film. (b) Evolution of MSD in the thick Co_3O_4 film as a function of pump-probe delay time. The pump wavelength is 700 nm and the probe wavelength is 570 nm. Error bars represent 2D Gaussian fitting errors.

Supplementary Note 7

Effect of Blue Shift on Hole Transport Measurements

In ultrafast optical nanoscopy measurements, the choice of probe wavelength determines the photo-induced species monitored. The precise single-wavelength probing enables direct access to the spatiotemporal evolution of specific photo-induced species, but spectral shifts across a wide spectral range can potentially impact single-wavelength ultrafast optical nanoscopy measurements.

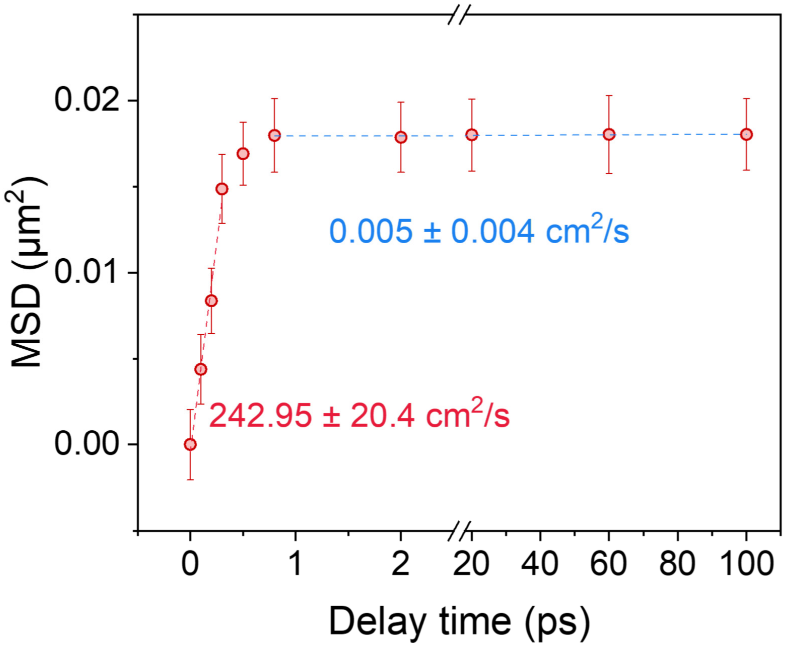

Supplementary Figure 13: Evolution of variance of MSD as a function of pump-probe delay time at a 700 nm pump and a 590 nm probe. The corresponding diffusion constant is included. Error bars represent 2D Gaussian fitting errors.

Thus, to exclude artifacts caused by spectral shifts, a 590 nm wavelength was chosen instead of the primarily used 570 nm probe wavelength in this study (which was used for monitoring the transport of hot holes and small hole polarons). The results, shown in Supplementary Figure 13, indicate that compared to the 570 nm probe wavelength, the 590 nm probe is closer to the higher energy holes (hot holes not fully cooled to the band edge) rather than the small hole polaron states after the hot holes are cooled[10]. Therefore, the transport of hot holes observed under the 590 nm probe occurs in less than 1 ps, with a slightly higher transport rate. This aligns with the cooling behavior of

hot holes, which cool primarily by emitting longitudinal optical (LO) phonons. Concurrently, the kinetic energy of hot holes decreases due to hole-phonon scattering, gradually cooling to the band edge. The 570 nm probe monitors the transport of fully cooled hot holes, while the 590 nm probe only monitors the transport of the initially hot holes (i.e., not yet cooled) and thus observed a shorter hot hole transport duration and a larger transport rate.

Supplementary Note 8

2D Maps of Co_3O_4 at Different Pump Wavelengths

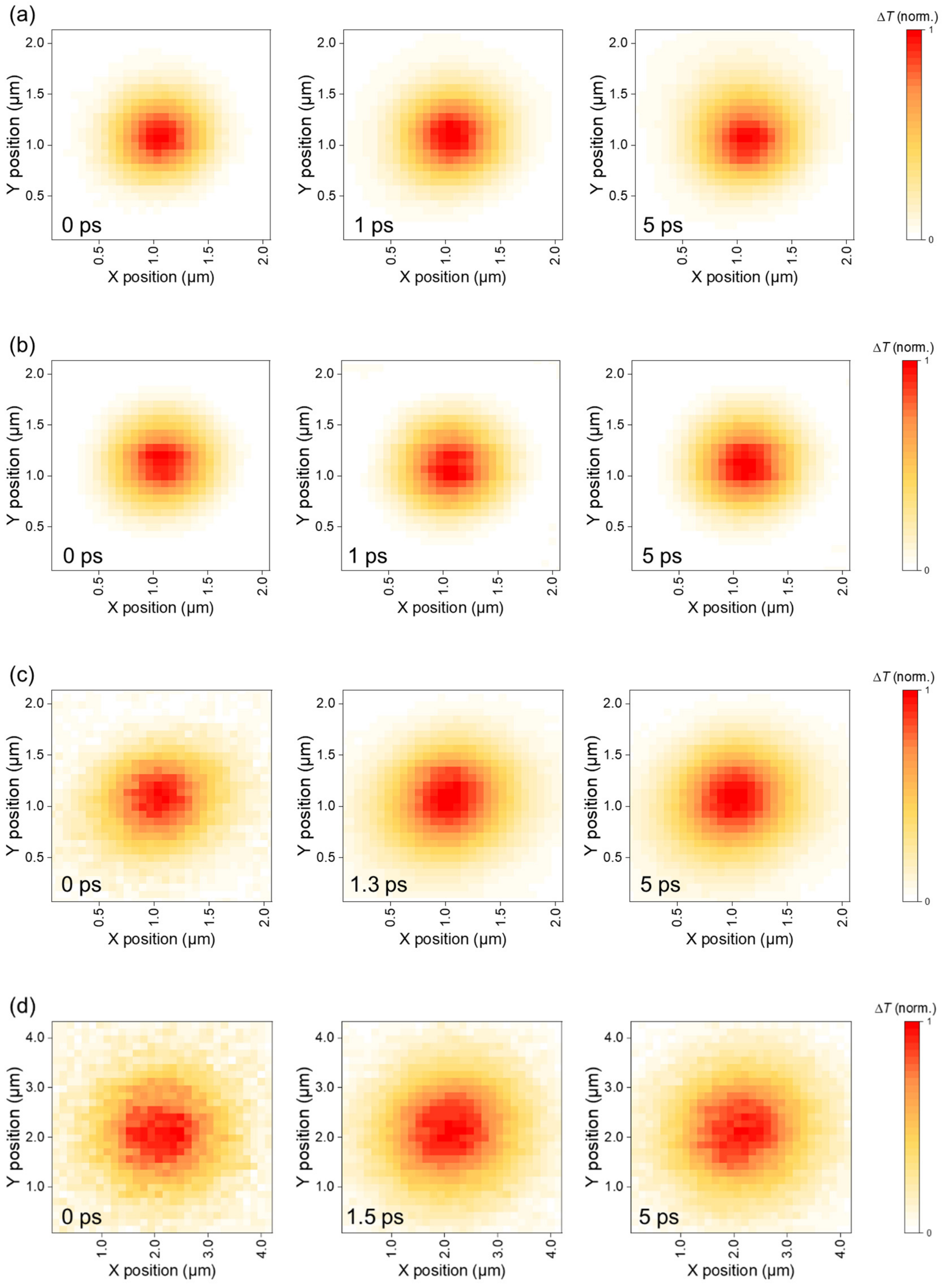

Supplementary Figure 14: 2D spatial maps of ultrafast optical nanoscopy images of Co_3O_4 at representative time delays under pump photon energies of (a) 2.58 eV, (b) 2.30 eV, (c) 1.94 eV, and (d) 1.55 eV.

Supplementary Note 9

Calculation of Hole Diffusion Length

The hole diffusion length can be calculated using Equation *S1*:

$$L = \sqrt{\sigma_t^2 - \sigma_0^2} \qquad (S1)$$

Take 1.77 eV excitation as an example, the hot hole regime ends at 1 ps and we can get $L_{Hot\ hole}$ = 169 nm. For the polaron transport regime, by fitting the TA dynamics at 570 nm, the lifetime of small hole polaron is about 800 ps. Combined with the diffusion constant of hopping regime (0.005 cm^2/s), we can get $L_{polaron}$ = 28.3 nm. In addition, we can also calculate the $L_{polaron}$ by using Equation *S1*. Supplementary Figure 15 shows the ultrafast nanoscopy profile with long delay time. By using $\sigma^2_{t2=1000\,ps}$ and $\sigma^2_{t1=1\,ps}$, we can get $L_{polaron}$=27.2 nm, which is in perfect agreement with the previous calculation scheme.

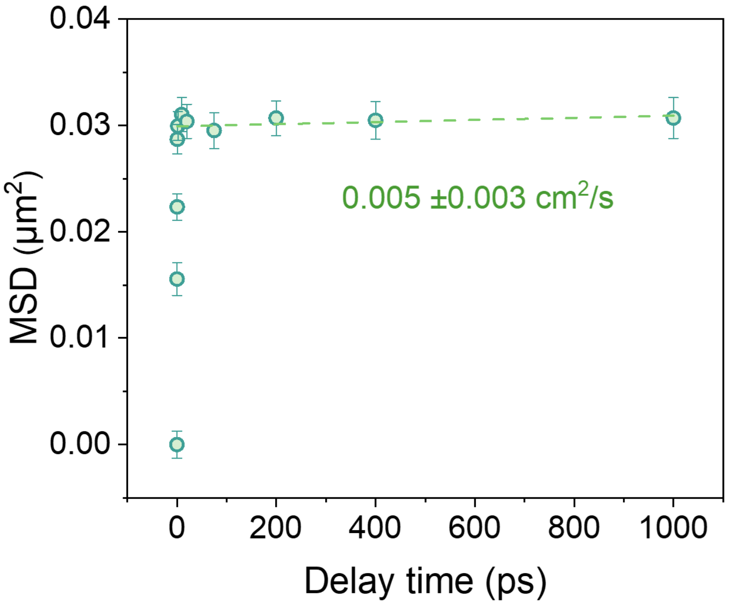

Supplementary Figure 15: Long timescale ultrafast nanoscopy profile. Pump: 1.77 eV. Error bars represent 2D Gaussian fitting errors.

The uncertainty in the extracted diffusion length can be evaluated through a sensitivity analysis by differentiating Equation S1, yielding:

$$\Delta L = \sqrt{\frac{\sigma_t^2}{\sigma_t^2 - \sigma_0^2}(\Delta\sigma_t)^2 + \frac{\sigma_0^2}{\sigma_t^2 - \sigma_0^2}(\Delta\sigma_0)^2} = \sqrt{\Delta\sigma_t^2 + \left(\frac{\sigma_0}{L}\right)^2 (\Delta\sigma_0^2 - \Delta\sigma_t^2)} \quad (S2)$$

Equation S2 shows that the uncertainty in diffusion length arises from the errors in the measured Gaussian variances at different time delays ($\Delta\sigma_t$ and $\Delta\sigma_0$), as well as the ratio between the initial beam width and the total transport length ($\frac{\sigma_0}{L}$).

To quantify this, we performed error analysis using three representative pump-probe delays from our dataset. The corresponding raw spatial data, two-dimensional Gaussian fitting results, and residuals are shown in the Supplementary Figure 16.

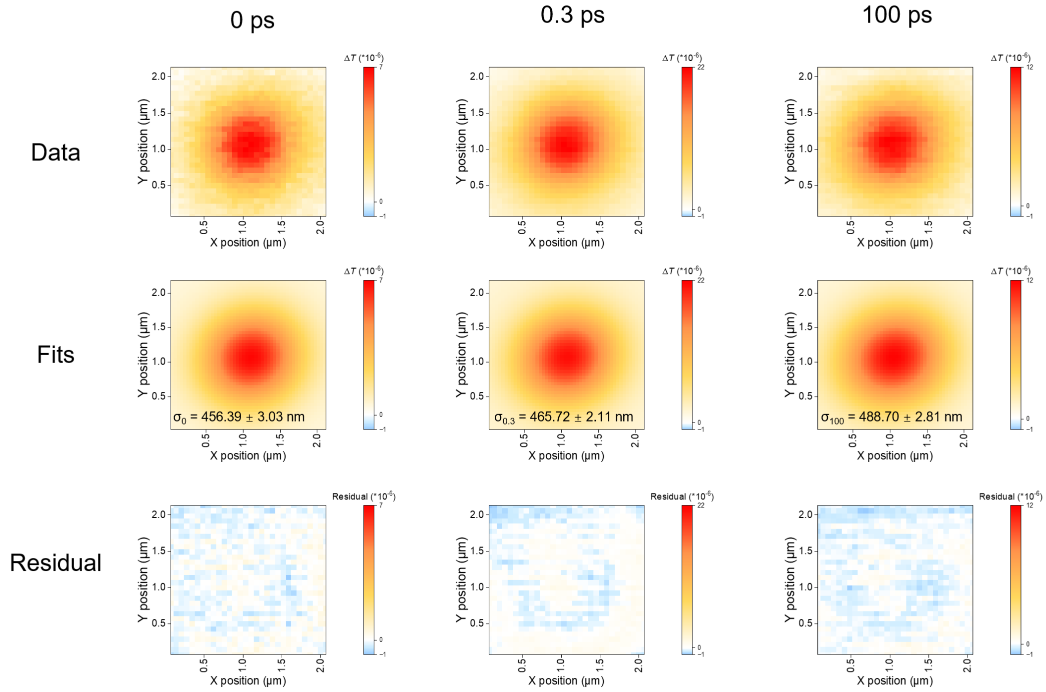

Supplementary Figure 16: The raw data, fitting results and residuals of Co_3O_4 at 1.77 eV pump with representative time delays.

Based on this analysis, the uncertainties in the extracted transport lengths at 0.3 ps and 100 ps are estimated to be 18.33 nm and 11.14 nm, respectively.

Supplementary Note 10

Origin of the Photoconductivity Decay

In TMOs, hot carrier relaxation competes between self-trapping into low mobility small polaron states and cooling via longitudinal optical (LO) phonon emission. As discussed in the main text, self-trapping is often dominant and leads to a rapid drop in photoconductivity. To examine its role here, we compared normalized THz dynamics at different pump photon energies (Supplementary Figure 17) and found nearly identical decay traces. This pump photon energy independence contrasts with the slower cooling expected for higher excess energies, supporting the scenario in which self-trapping occurs before full carrier cooling, which is consistent with previous temperature-dependent TA studies[11]. These observations indicate that the initial photoconductivity decay is primarily governed by ultrafast carrier localization, rather than by phonon-mediated cooling dynamics.

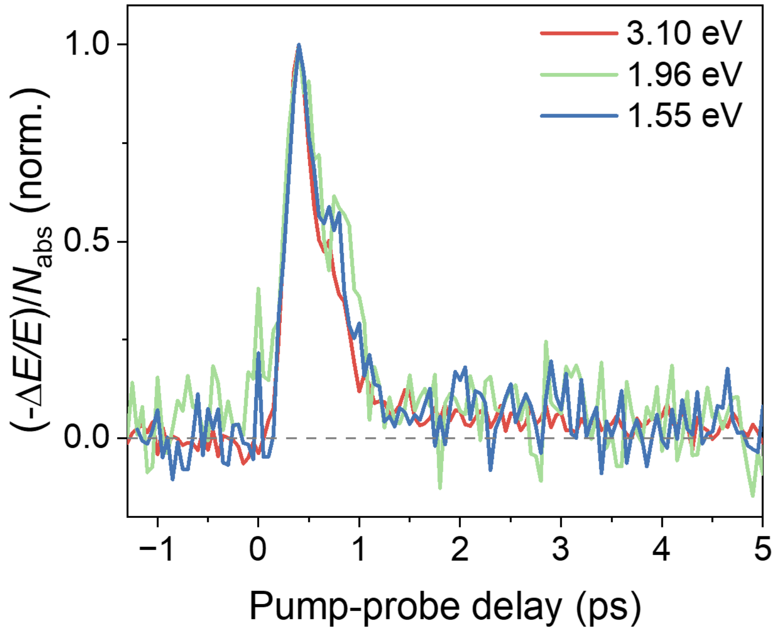

Supplementary Figure 17: Normalized photoconductivity dynamics of Co_3O_4 at different pump photon energies.

Supplementary Note 11

Comparison of Carrier Probing: Ultrafast Optical Nanoscopy vs. THz Spectroscopy

While the dynamics observed in the ultrafast optical nanoscopy (Figure 2c) and OPTP measurements (Figure 3b) agree well, the response to varying excitation photon energies (Supplementary Figure 18) is different for OPTP. This can be traced to OPTP's sensitivity to both electrons and holes, whereas nanoscopy allows for the selective monitoring of hole transport. The THz response is also sensitive to electrons that have a lower effective mass than the holes. Thus, in our THz measurements, when the pump photon energy is increased, the photoconductivity rises due to (1) the greater photoconductivity weight of electrons relative to holes[12], and (2) the stronger transport capability of hot electrons compared to band-edge cold electrons[13, 14].

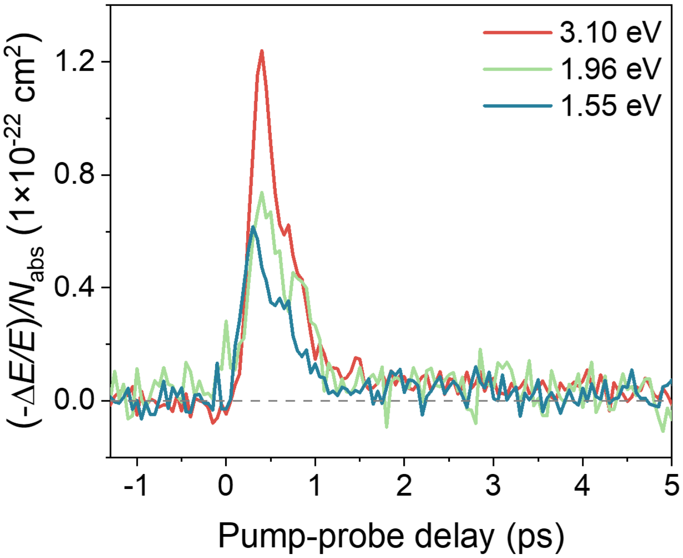

Supplementary Figure 18: Pump wavelength-dependent photoconductivity dynamics.

Under equilibrium or near-equilibrium conditions, carrier transport properties are often described using the Einstein relation, which links the diffusion coefficient (D) and mobility (μ):

$$\frac{D}{\mu} = \frac{k_B T}{q} \qquad (S3)$$

Here, k_B is the Boltzmann constant, T is the absolute temperature, and q is the elementary charge.

This relation allows for a direct comparison between carrier diffusion and mobility. However, in the hot carrier transport regime, where carriers possess excess energy and their distribution significantly deviates from thermal equilibrium, the assumptions underlying the Einstein relation break down. The carrier system is no longer governed by a Boltzmann distribution, and energy relaxation, nonthermal scattering, and transient localization effects play dominant roles in determining transport behavior.

Consequently, while both probes can provide charge transport information, a quantitative comparison between the diffusion constants obtained from ultrafast optical nanoscopy and carrier mobilities derived from THz spectroscopy may not always be valid. Ultrafast optical nanoscopy, with its high spatial and temporal resolution, enables direct visualization of the diffusion behavior of photogenerated carriers, capturing their spatial evolution on fs to ns timescales. In contrast, THz spectroscopy is sensitive to the collective dielectric response of the material at low frequencies (meV range), and provides quantitative access to short-range carrier mobility, momentum scattering rates, and their associated transport mechanisms.

While these techniques probe fundamentally different aspects of carrier dynamics—macroscopic spatial diffusion versus microscopic, frequency-resolved conductivity—they are inherently complementary. Ultrafast optical nanoscopy reveals how carriers propagate through the material over time and space, reflecting the cumulative effects of all underlying transport processes. THz spectroscopy, on the other hand, captures the THz response of the carrier, offering insight into intrinsic carrier mobility and scattering dynamics. The synergistic integration of ultrafast optical nanoscopy with THz spectroscopy establishes a complementary analytical framework for deciphering multiscale carrier dynamics in TMOs.

Supplementary Note 12

Extracting Complex Photoconductivity in TMOs

In our experimental configuration, the photoinduced conductivity decays with a lifetime that is significantly shorter than the temporal extent of the probing THz pulse. Standard THz time-domain spectroscopy, which involves scanning the delay of the sampling beam alone, operates under a quasi-steady-state assumption. This method presumes the photoconductivity is effectively constant over the duration of the THz probe. For fast decaying signals, the approach introduces complications for frequency-resolved photoconductivity (Figure 3d) because the leading and trailing portions of the THz pulse probe different instantaneous carrier densities.

To circumvent this limitation, we implemented an alternative detection strategy. In this modified scheme, the temporal delay between the pump (for photoexcitation) and the sampling pulse (for electro-optic detection) was held constant. Instead, their respective delay stages were translated in unison relative to the THz pulse. This synchronization guarantees that the entire THz waveform probes an identical, non-equilibrium state of the carrier population. The result is a measurement that is functionally equivalent to scanning the delay of the THz pulse against a fixed, transient photoconductivity. Consequently, this method yields more reliable, frequency-resolved complex conductivity spectra.

The samples studied in this work have thicknesses of sub-μm, which is significantly smaller than the typical wavelength of THz radiation (~300 μm). Given their extremely thin nature, we applied the thin-film approximation to obtain their frequency-dependent complex conductivity $\sigma(\omega)$, given by[15, 16]:

$$\Delta\sigma(\omega) = -\frac{n_{\mathrm{sub}} + n_{\mathrm{air}}}{Z_0 l}\left(\frac{\Delta E}{E}\right) \qquad (S4)$$

Here, Z_0=377 Ω is the impedance of free space, n_{sub}= 1.96 is the THz refractive index of fused silica substrate, $n_{\mathrm{air}} \approx 1$ is the refractive index of the air, and l is the thickness of the sample.

As mentioned in the main text, the relative attenuation of the pump-induced terahertz

electric field ($-\Delta E/E$) is proportional to photoconductivity ($\sigma = ne\mu$). During THz measurements at different temperatures, the sample's absorption of light changes significantly with temperature, causing variations in the density of photocarriers n, which in turn affects the accurate quantification of mobility μ.

For that, we have calibrated the absorption changes at different T. By knowing the absorption photon density, we have further converted photoconductivity to the product of the free carrier generation quantum yield and carrier mobility ($\phi\mu$), given by[12]:

$$\frac{\sigma}{N_{\mathrm{abs\_vol}}} = \left(-\frac{n_{\mathrm{sub}} + n_{\mathrm{air}}}{Z_0 l}\frac{\Delta E}{E}\right)\frac{l}{N_{\mathrm{abs}}} = \frac{ne\mu}{N_{\mathrm{abs\_vol}}} \propto \phi\mu \qquad (S5)$$

where N_{abs} is the density of absorbed photons per area, obtained from the product of the incident photon density and the percentage of absorbance at different temperatures. The $N_{\mathrm{abs\_vol}}$ is the number of absorbed photons per volume. ϕ is the free carrier generation quantum yield, which is $n = \phi \cdot N_{\mathrm{abs\_vol}}$. So, we have:

$$\phi\mu \propto \left(-\frac{\Delta E}{E}\right)\left(\frac{n_{\mathrm{sub}} + n_{\mathrm{air}}}{Z_0}\right)\left(\frac{1}{e \cdot N_{\mathrm{abs}}}\right) \qquad (S6)$$

Supplementary Note 13

Temperature-Dependent Photoconductivity and Associated Microscopic Transport Parameters

As shown in Figure 3c, while the $\phi\mu$ product in the hot carrier transport regime increases at lower temperatures, it decreases in the polaron hopping transport regime. We note that the free carrier generation quantum yield (ϕ) may exhibit temperature dependence, which could also influence the analysis of mobility (μ)[17, 18].

Following the Drude-Smith model (as described in Equation 1 of the main text), we can infer the plasma frequency ω_P, which corresponds to the resonant frequency of the plasma oscillations of free charge carriers. This frequency serves as a critical parameter that reflects the collective oscillation of free carriers in response to an external electric field. Importantly, the plasma frequency is directly proportional to the square root of the free carrier density within the material, given by:

$$\omega_P = \sqrt{\frac{e^2 n}{\varepsilon_0 m^*}} \qquad (S7)$$

And:

$$n \propto {\omega_P}^2 \qquad (S8)$$

Supplementary Figure 19 (a) shows N_{abs} and ${\omega_P}^2$ (The square of the plasma frequency, representing the quantity of free charge carriers) at different temperatures. The temperature dependence of ${\omega_P}^2$ is more pronounced, indicating a lower number of free carriers at lower temperatures. We use ${\omega_P}^2/N_{\text{abs}}$ as a phenomenological representation of the photon-to-free-carrier conversion yield ϕ. As shown in Supplementary Figure 19 (b), the ϕ decreases with decreasing temperature.

On the other hand, $\phi\mu$ within the hot carrier transport regime is found to increase with lowering temperatures, indicating that μincreases at lower temperature. This result further confirms the band-like nature of hot carrier transport.

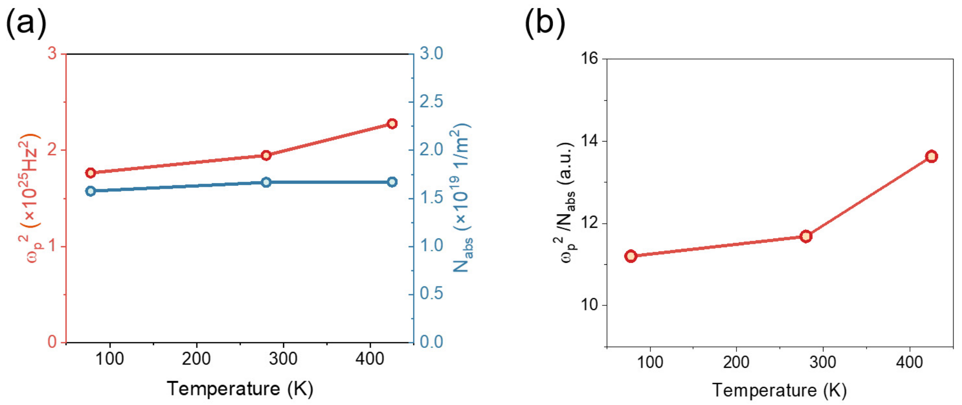

Supplementary Figure 19: (a) N_{abs} and ${\omega_P}^2$ at different temperatures. (b) Photon-free carrier conversion yield at different temperatures.

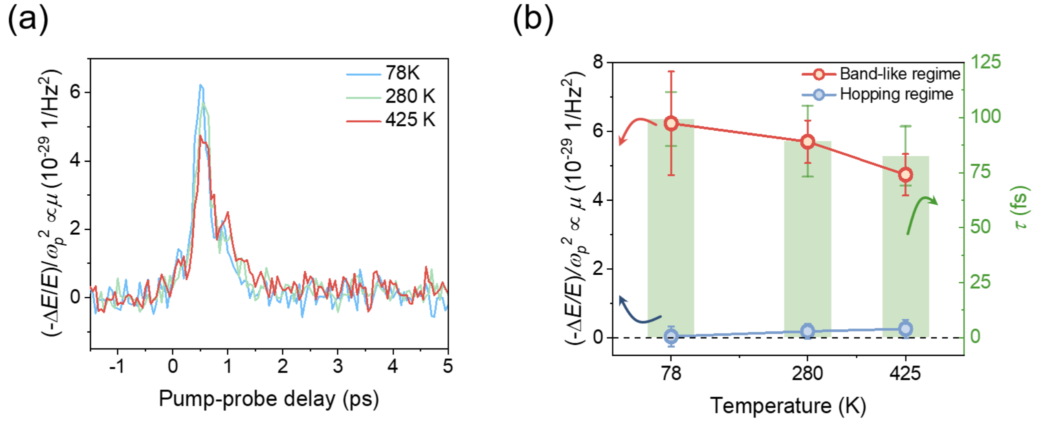

Supplementary Figure 20: (a) The carrier mobility at different temperatures was obtained by dividing the relative attenuation of the terahertz electric field by the plasma frequency. (b) Carrier mobility in the band-like regime (red) and hopping regime (blue) and the inferred charge scattering time (green) from the Drude-Smith model. Error bars represent standard deviations from 300 scans (hot-carrier transport), standard deviations of 2–5 ps averaged values (polaron hopping), and fitting uncertainties from the Drude–Smith model (charge scattering time).

Based on equation S5 and S8, we get:

$$(-\Delta E/E)/{\omega_P}^2 \propto \mu \qquad (S9)$$

$(-\Delta E/E)/{\omega_P}^2$is directly related to μ. For that, we further plot temperature dependent $(-\Delta E/E)/{\omega_P}^2$ as shown in Supplementary Figure 20. As expected, the hot carrier mobility at 78 K is 31% higher than at 425 K. In the polaron transport regime, we still observe a 5.6-fold increase in carrier mobility at 425 K compared to 78 K, further confirming thermally activated hopping transport of polaron.

Supplementary Note 14

Steady State Absorption of α-Fe_2O_3 Thin Film

Similar to Co_3O_4, the absorption spectra of α-Fe_2O_3 thin film were fitted using a multimodal Gaussian model. The fitting parameters refer to the assignment of Hayes et al.[8], and the fitting results are shown in Supplementary Table 2.

Supplementary Table 2: Optical transition assignments for α-Fe_2O_3

Peak	Energy (eV)	Intensity (arb. units)	Linewidth (FWHM, eV)
LMCT 1	5.51	1	2.81
LMCT 2	3.11	0.111	0.78
MMT 1	2.32	0.004	0.21
MMT 2	2.47	0.003	0.24
MMT 3	2.81	0.041	0.69
MMT 4	3.89	0.035	0.52
MMT 5	4.48	0.022	0.58

To be more specific, in previous reports, the electron transition near the absorption edge (i.e., MMT 1) in hematite is usually attributed to the local ligand field (LF) transition within the Fe^{3+} ion. LF transitions are intrinsically intrametallic (i.e., occur within the d orbitals of the same metal center) and do not involve net charge transfer, in contrast to inter-site metal-to-metal charge transfer (MMCT) and ligand-to-metal charge transfer (LMCT) transitions.

The strong localization of LF transitions restricts the spatial extent of the resulting excited-state wavefunctions, thereby limiting the mobility of the associated carriers. This provides a physically reasonable explanation for the lower diffusivity observed under low-photon-energy excitation (2.13 eV), where LF transitions dominate the optical absorption.

However, the spectral assignment of absorption features at higher photon energies, especially those related to pair LF transitions, remains controversial. The pair LF transitions involve simultaneous LF transitions on adjacent Fe sites and have been reported over a wide energy range (~ 3-5.6 eV)[19-21]. Their spectral features significantly overlap with those of LMCT transitions, making unambiguous attribution challenging.

Su et al. proposed that pair LF transitions may lead to the formation of Fe^{2+} - Fe^{4+} charge pairs via superexchange or hopping mechanisms, thereby imparting partial charge-transfer character, even though LF transitions themselves do not directly involve inter-site charge transfer[22]. In addition, Chernyshova et al. pointed out that high-energy LF transitions, when mixed with delocalized LMCT absorption, may exhibit partial delocalization, which further obscures their localized nature and complicates precise spectral assignments[21]. Moreover, due to selection rules, the relative contribution of LF transitions tends to diminish at higher excitation energies, where LMCT transitions dominate the absorption cross-section.

In light of these considerations, we did not attempt a quantitative deconvolution of LF and LMCT contributions in the high-energy spectral region. Instead, we focused our analysis on the correlation between photon-energy-dependent excitation pathways and the observed trends in hot carrier transport.

Supplementary Note 15

Transient Absorption (TA) of α-Fe_2O_3 Thin Film

TA measurements were performed on α-Fe_2O_3 film using a pump photon energy of 2.48 eV, with the results shown in Supplementary Figure 21. According to electrochemical spectroscopy measurements by Barroso et al., the broad absorption feature around 675 nm corresponds to hole filling in the valence band[23].

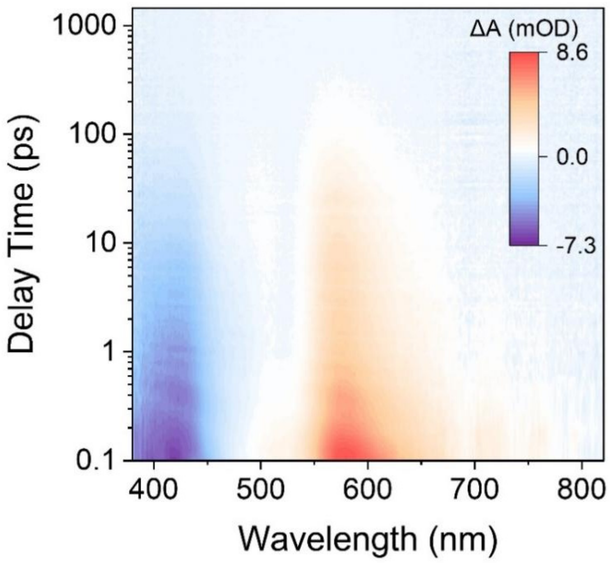

Supplementary Figure 21: 2D color plot of TA spectra of α-Fe_2O_3 film at 2.48 eV pump photon energy.

Supplementary Note 16

Impact of Higher-Order Recombination on Ultrafast Optical Nanoscopy Measurements

Liao et al. revealed Auger recombination of self-trapped excitons (STE) in α-Fe_2O_3 through TA measurements. We note that higher-order processes may cause artificial broadening in ultrafast optical nanoscopy measurements, leading to an overestimation of the diffusion constant.

In ultrafast optical nanoscopy measurements, the Gaussian pump beam is used to excite the sample, resulting in a spatially Gaussian distribution of photocarriers. If the photocarrier is in diffusive transport, then the carrier population as a function of space and time can be described by the following equations, including decay and diffusion:

$$\frac{\partial n(x,y,t)}{\partial t} = D\left[\frac{\partial^2 n(x,y,t)}{\partial x^2} + \frac{\partial^2 n(x,y,t)}{\partial y^2}\right] - \frac{n(x,y,t)}{\tau} - \gamma n^2(x,y,t) - \eta n^3(x,y,t) \quad (S10)$$

Here, the carrier population $n(x,y,t)$ could be approximated to a Gaussian distribution, D is the diffusion constant, τ is the carrier lifetime, γ is the bimolecular recombination coefficient and η is the Auger recombination coefficient.

When higher-order recombination terms are negligible, the equation can be simplified to:

$$\frac{\partial n(x,y,t)}{\partial t} = D\left[\frac{\partial^2 n(x,y,t)}{\partial x^2} + \frac{\partial^2 n(x,y,t)}{\partial y^2}\right] - \frac{n(x,y,t)}{\tau} \quad (S11)$$

This leads to:

$$D = \frac{\sigma_t^2 - \sigma_0^2}{2t} = \frac{MSD}{2t} \quad (S12)$$

However, the power-dependent dynamics of hematite (Supplementary Figure 22) indicate that when the pump fluence exceeds 0.3 mJ/cm², higher-order recombination

becomes significant, leading to a nonlinear dependence of the annihilation rate on local carrier density. Carriers in the central region recombine faster than those at the edges, resulting in an artificial broadening of σ_t^2 obtained by two-dimensional Gaussian fitting.

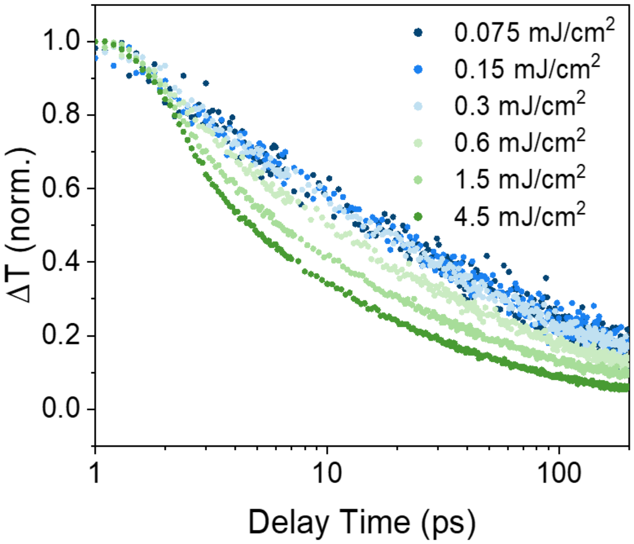

Supplementary Figure 22: Carrier dynamics of hematite thin films pumped at 410 nm and probe at 675 nm for different excitation fluence.

We performed ultrafast optical nanoscopy measurements at a laser fluence of 1.5 mJ/cm^2, and the relationship between MSD and delay time is sublinear, as shown in Supplementary Figure 23. To avoid overestimation of the carrier diffusion constant, all ultrafast optical nanoscopy measurements reported in the main text were conducted with photocarrier densities below this threshold.

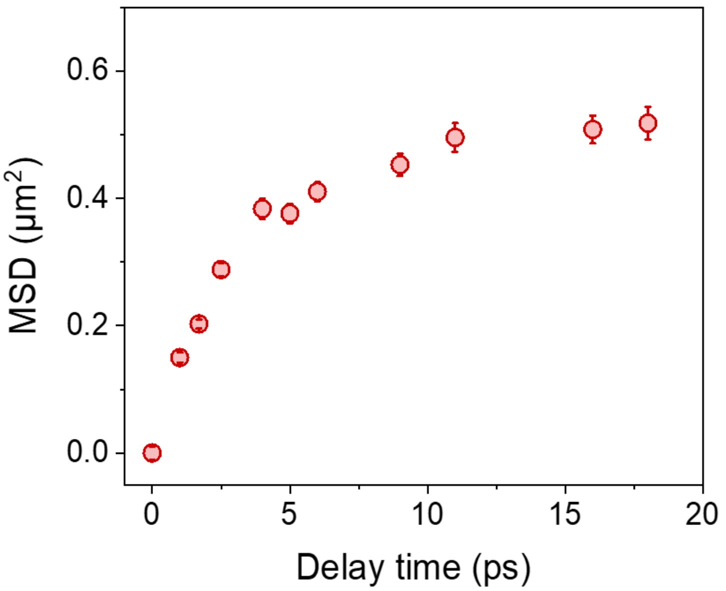

Supplementary Figure 23: The evolution of MSD in hematite under higher-order recombination as a function of delay time. The pump wavelength is 410 nm and the pump fluence is 1.5 mJ/cm^2. Due to the broadening of the Gaussian distribution caused by nonlinear recombination processes, the increase in MSD cannot be considered to be contributed by carrier diffusion alone. Error bars represent 2D Gaussian fitting errors.

Supplementary Note 17

2D Maps of hematite at different pump wavelengths

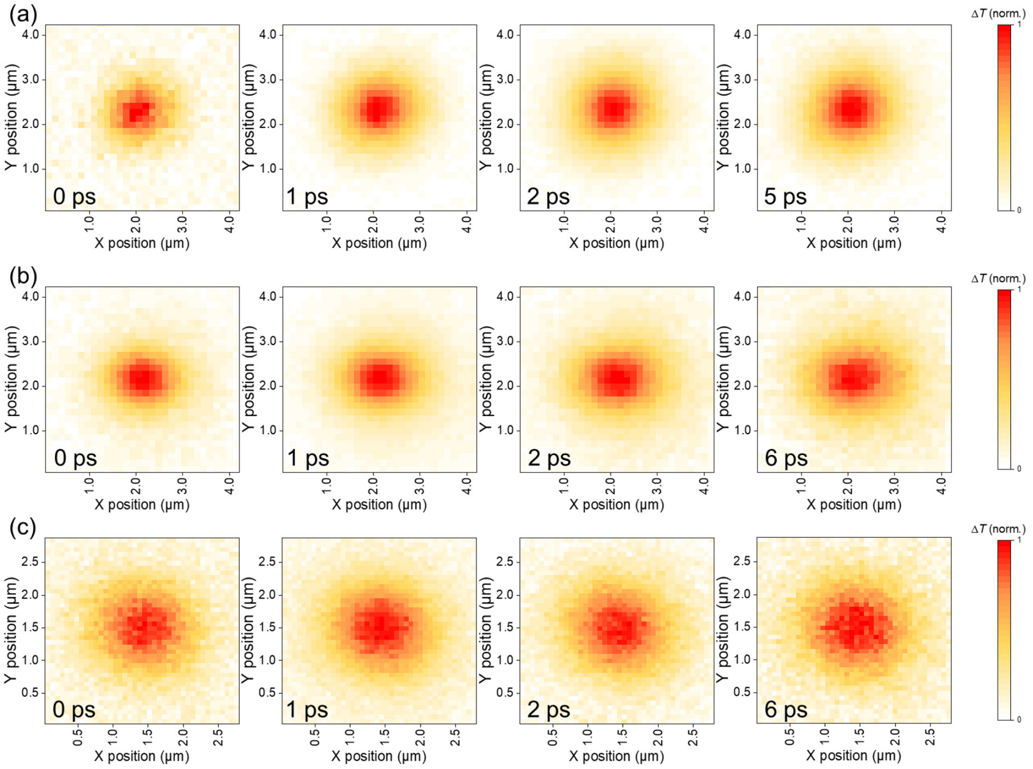

Supplementary Figure 24: 2D spatial maps of ultrafast optical nanoscopy images of hematite at representative time delays under pump photon energies of (a) 3.02 eV, (b) 2.53 eV, and (c) 2.13 eV.

Supplementary Note 18

Numerical Simulation of Carrier Extraction Dynamics in α-Fe_2O_3 Photoelectrode

To further evaluate the contribution of hot carriers to photocurrent generation in PEC systems, we developed a one-dimensional carrier transport model that quantitatively describes the spatiotemporal dynamics of both hot and cold carrier populations under photoexcitation. This model incorporates wavelength-dependent optical absorption, carrier diffusion, recombination, and the quantum yield of delocalized (hot) carriers.

The initial spatial distribution of photogenerated carriers follows the Beer-Lambert law:

$$n(z, t = 0) \propto e^{-\alpha z} \qquad (S13)$$

Here, z denotes the depth coordinate measured from the illuminated surface ($z = 0$) to the collecting interface ($z = L$, where L is the thickness of the film), and t is the time after photoexcitation.

Based on the hot carrier quantum yield (QY), determined from the LMCT contribution in Figure 4a, the total carrier population is partitioned into hot and cold components:

$$n_{\mathrm{hot}}(z, 0) = QY \cdot n(z, 0) \qquad (S14)$$

$$n_{\mathrm{cold}}(z, 0) = (1 - QY) \cdot n(z, 0) \qquad (S15)$$

The temporal evolution of each carrier species is governed by diffusion, recombination, and hot carriers cooling (then convert to cold carriers):

$$\frac{\partial n_{\mathrm{hot}}}{\partial t} = D_{\mathrm{hot}} \frac{\partial^2 n_{\mathrm{hot}}}{\partial z^2} - \frac{n_{\mathrm{hot}}}{\tau_{\mathrm{hot}}} \qquad (S16)$$

$$\frac{\partial n_{\mathrm{cold}}}{\partial t} = D_{\mathrm{cold}} \frac{\partial^2 n_{\mathrm{cold}}}{\partial z^2} - \frac{n_{\mathrm{cold}}}{\tau_{\mathrm{cold}}} + \frac{n_{\mathrm{hot}}}{\tau_{\mathrm{hot}}} \qquad (S17)$$

The last term in the cold carrier equation accounts for self-trapping of hot carriers. D_{hot}, τ_{hot}, $D_{\mathrm{cold}}$and τ_{cold} represent the diffusion constant of hot carriers, the cooling time constant of hot carriers, the diffusion constant of cold carriers (polarons) and the

lifetime of polarons. Their values are all taken from the parameters extracted in this work.

Carrier extraction is modeled by evaluating the net diffusive flux at the collecting interface ($z = L$).

$$J(t) = -D_{\mathrm{hot}} \frac{\partial n_{\mathrm{hot}}}{\partial z}\bigg|_{z=L} - D_{\mathrm{cold}} \frac{\partial n_{\mathrm{cold}}}{\partial z}\bigg|_{z=L} \qquad (S18)$$

The extraction efficiency is then defined as:

$$\eta_{\mathrm{ext}} = \frac{1}{N_0} \int_0^T J(t) dt \qquad (S19)$$

Where T is the total simulation time and N_0 is the total initial carrier population in the film:

$$N_0 = \int_0^L [n_{\mathrm{hot}}(z, 0) + n_{\mathrm{cold}}(z, 0)] dz \qquad (S20)$$

To quantify the role of ultrafast carrier extraction, two characteristic metrics are defined:

(1) Early-time extraction efficiency, calculated by integrating $J(t)$ over the first 2 ps, which represents the fraction of hot carriers extracted before cooling and self-trapping;

(2) Total extraction efficiency, integrated over the full simulation duration (1,500 ps), which reflects the cumulative collection yield over the carrier lifetime.

We conducted simulations using a 500 nm thick hematite film, and the results are shown in Supplementary Figure 25.

Under 2.53 eV excitation, the relatively long photon penetration depth places the initial carrier distribution closer to the collecting interface (Supplementary Figure 25a). However, due to the dominant contribution of localized transitions at this wavelength, we estimate that only ~61% of carriers are delocalized and thus able to participate in hot carrier transport. The corresponding early-time and total extraction efficiencies are 1.54% and 2.15%, respectively.

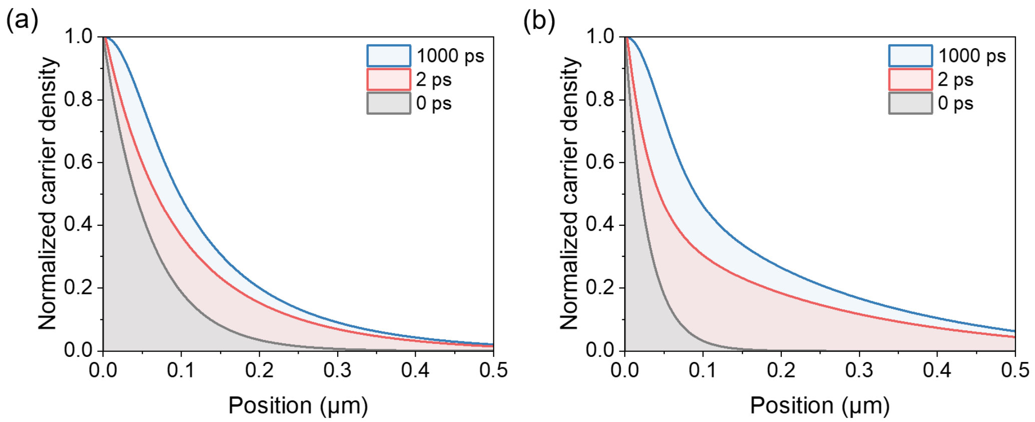

Supplementary Figure 25: Simulated spatial distribution of photogenerated carriers in a 0.5 μm thick hematite film at different time delays under excitation with photon energies of (a) 2.53 eV and (b) 3.02 eV. The excitation surface is located at x = 0, and the collecting interface is at x = 0.5 μm.

In contrast, under 3.02 eV excitation, although the photon penetration depth is shorter, the hot carrier quantum yield increases significantly (~84%), and the diffusion coefficient of delocalized carriers is also larger (Supplementary Figure 25b). Consequently, the early-time extraction efficiency reaches 8.07%, and the total extraction efficiency increases to 9.46%. These results are consistent with the higher photocurrent under high-energy excitation reported by Kay et al[24].

It is important to note that the diffusion-based model employed here is idealized and does not account for additional effects such as drift currents induced by applied bias, electric field-enhanced charge separation, or losses due to interfacial recombination and bulk defects. Nonetheless, the simulations indicate that hot carriers can play a significant role in photocurrent generation, particularly under above-bandgap excitation. The existence of a fast extraction channel mediated by ultrafast hot carrier transport underscores the potential relevance of hot carrier dynamics for optimizing PEC device performance.

Supplementary Note 19

Ultrafast optical nanoscopy measurement of Cu_2O

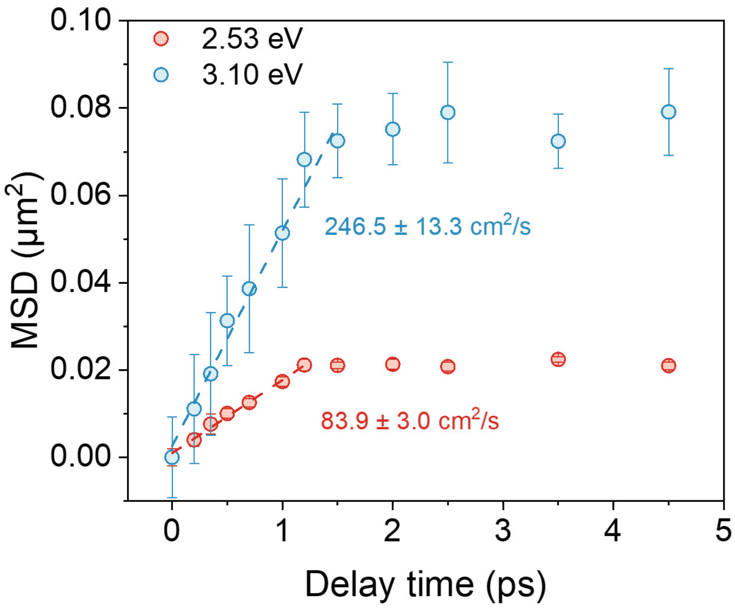

Supplementary Figure 26: MSD evolution of Cu_2O at 2.53 eV and 3.1 eV excitations with delay time. The probe wavelength is 500 nm. Error bars represent 2D Gaussian fitting errors.

Supplementary references

1. Hadjiev V, Iliev M, Vergilov I. The raman spectra of Co3O4. *Journal of Physics C: Solid State Physics* **21**, L199 (1988).
2. Lorite I, Romero J, Fernández J. Effects of the agglomeration state on the Raman properties of Co3O4 nanoparticles. *Journal of Raman Spectroscopy* **43**, 1443-1448 (2012).
3. Qiao L*, et al.* Nature of the band gap and origin of the electro-/photo-activity of Co 3 O 4. *Journal of Materials Chemistry C* **1**, 4628-4633 (2013).
4. Waegele MM, Doan HQ, Cuk T. Long-Lived Photoexcited Carrier Dynamics of d–d Excitations in Spinel Ordered Co3O4. *The Journal of Physical Chemistry C* **118**, 3426-3432 (2014).
5. Schmidt R*, et al.* Ultrafast coulomb-induced intervalley coupling in atomically thin WS2. *Nano letters* **16**, 2945-2950 (2016).
6. Wang L*, et al.* Phonon-driven transient bandgap renormalization in perovskite single crystals. *Materials horizons* **10**, 4192-4201 (2023).
7. Cooper JK, Reyes-Lillo SE, Hess LH, Jiang C-M, Neaton JB, Sharp ID. Physical origins of the transient absorption spectra and dynamics in thin-film semiconductors: the case of BiVO4. *The Journal of Physical Chemistry C* **122**, 20642-20652 (2018).
8. Hayes D*, et al.* Electronic and nuclear contributions to time-resolved optical and X-ray absorption spectra of hematite and insights into photoelectrochemical performance. *Energy & Environmental Science* **9**, 3754-3769 (2016).
9. Wang Y, Li K, Jiang L, Gao G, Li J, Zhu T. Regulation of Hot Electrons Transport Achieved through Controlled Electron‐Phonon Coupling in Metallic Heterostructures. *Small* **20**, 2400017 (2024).
10. Najafi E, Ivanov V, Zewail A, Bernardi MJNc. Super-diffusion of excited carriers in semiconductors. **8**, 15177 (2017).
11. Zhang Y, Zhang C, Huang X, Yang Z, Zhang KH, Yang Y. Barrierless Self-Trapping of Photocarriers in Co3O4. *The Journal of Physical Chemistry Letters* **12**, 12033-12039 (2021).
12. Giannini S*, et al.* Transiently delocalized states enhance hole mobility in organic molecular semiconductors. *Nature materials* **22**, 1361-1369 (2023).
13. Liu Q, Wei K, Tang Y, Xu Z, Cheng Xa, Jiang T. Visualizing Hot‐Carrier Expansion and Cascaded Transport in WS2 by Ultrafast Transient Absorption Microscopy. *Advanced Science* **9**, 2105746 (2022).
14. Deng S, Blach DD, Jin L, Huang L. Imaging carrier dynamics and transport in hybrid perovskites with transient absorption microscopy. *Advanced Energy Materials* **10**, 1903781 (2020).
15. Tinkham MJPR. Energy gap interpretation of experiments on infrared transmission through superconducting films. **104**, 845 (1956).
16. Nienhuys H-K, Sundström VJPRBCM, Physics M. Intrinsic complications in the analysis of optical-pump, terahertz probe experiments. **71**, 235110 (2005).
17. Ghosh S*, et al.* Band-like transport of charge carriers in oriented two-

dimensional conjugated covalent organic frameworks. **34**, 736-745 (2022).

18. Wang M*, et al.* Exceptionally high charge mobility in phthalocyanine-based poly (benzimidazobenzophenanthroline)-ladder-type two-dimensional conjugated polymers. **22**, 880-887 (2023).
19. He Y*, et al.* Size and structure effect on optical transitions of iron oxide nanocrystals. *Physical Review B—Condensed Matter and Materials Physics* **71**, 125411 (2005).
20. Liao P, Carter EA. Optical excitations in hematite (α-Fe2O3) via embedded cluster models: a CASPT2 study. *The Journal of Physical Chemistry C* **115**, 20795-20805 (2011).
21. Chernyshova I, Ponnurangam S, Somasundaran P. On the origin of an unusual dependence of (bio) chemical reactivity of ferric hydroxides on nanoparticle size. *Physical Chemistry Chemical Physics* **12**, 14045-14056 (2010).
22. Su Z, Baskin JS, Zhou W, Thomas JM, Zewail AH. Ultrafast elemental and oxidation-state mapping of hematite by 4D electron microscopy. *Journal of the American Chemical Society* **139**, 4916-4922 (2017).
23. Barroso M, Pendlebury SR, Cowan AJ, Durrant JR. Charge carrier trapping, recombination and transfer in hematite (α-Fe 2 O 3) water splitting photoanodes. *Chemical Science* **4**, 2724-2734 (2013).
24. Kay A, Grave DA, Deo Malviya K, Ellis DS, Dotan H, Rothschild A. Wavelength dependent photocurrent of hematite photoanodes: reassessing the hole collection length. *The Journal of Physical Chemistry C* **121**, 28287-28292 (2017).
